# Type A Aortic Dissection with Antegrade Intimointimal Intussusception

**DOI:** 10.14797/mdcvj.1210

**Published:** 2023-04-05

**Authors:** Christine Lannon, Priya Arunachalam, Lamees I. El Nihum, Nina Manian, Amr Telmesani, Qasim Al Abri, Michael J. Reardon

**Affiliations:** 1 Houston Methodist DeBakey Heart & Vascular Center, Houston Methodist Hospital, Houston, Texas, US; 2 Texas A&M College of Medicine, Bryan, Texas, US; 3 Baylor College of Medicine, Houston, Texas, US

**Keywords:** type A aortic dissection, intimointimal intussusception

## Abstract

We describe a 60-year-old man with a history of hypertension who presented to an outside emergency department with chest pain and left lower extremity numbness and weakness. Computed tomography (CT) revealed Stanford type A aortic dissection (TAAD), and he was transferred to our institution for emergent open surgical repair. Review of the outside CT showed no dissection flap in the ascending aorta and a complex flap in the proximal descending thoracic aorta consistent with complex intimal transection at the sinotubular junction and intimointimal intussusception. This case presents high-resolution diagnostic and intraoperative images and illustrates the importance of rapid diagnosis and recognition of the potentially complex nature of the aortic dissection to avoid impending hemodynamic deterioration.

## Introduction

Type A aortic dissection (TAAD) is an emergent vascular condition involving a tear in the intima of the ascending aorta. Blood flow into the media creates a false lumen that can progress along the aorta or rupture through the adventitia. Risk factors for TAAD include hypertension, dyslipidemia, connective tissue disorders, and an age range of 65 to 75 years.^1^ Patients with TAAD may present with sudden “tearing” chest pain that radiates to the back, neurological deficits, or syncope.^2^ The diagnosis can be made with computed tomography (CT) angiography or transesophageal echocardiogram (TEE), which allow visualization of the intimal tear, classification of the dissection, and assessment of the valve and arch involvement.^2^ Confirmed TAAD generally requires immediate surgical intervention to replace the damaged aorta with synthetic vascular graft and to assess the aortic valve.^2^ Endovascular stent graft repair of TAAD has been done but remains investigational.^3^ While the intimal tear in TAAD is usually transverse and comprises less than half of the aortic circumference, rare cases present with circumferential dissection and intimointimal intussusception, or “telescoping,” of the aorta, as described here.^4^

## Presentation

A 60-year-old male with a history of hypertension presented to an outside emergency department with chest pain and left lower extremity numbness and weakness.

## Investigations

**Figure 1:**
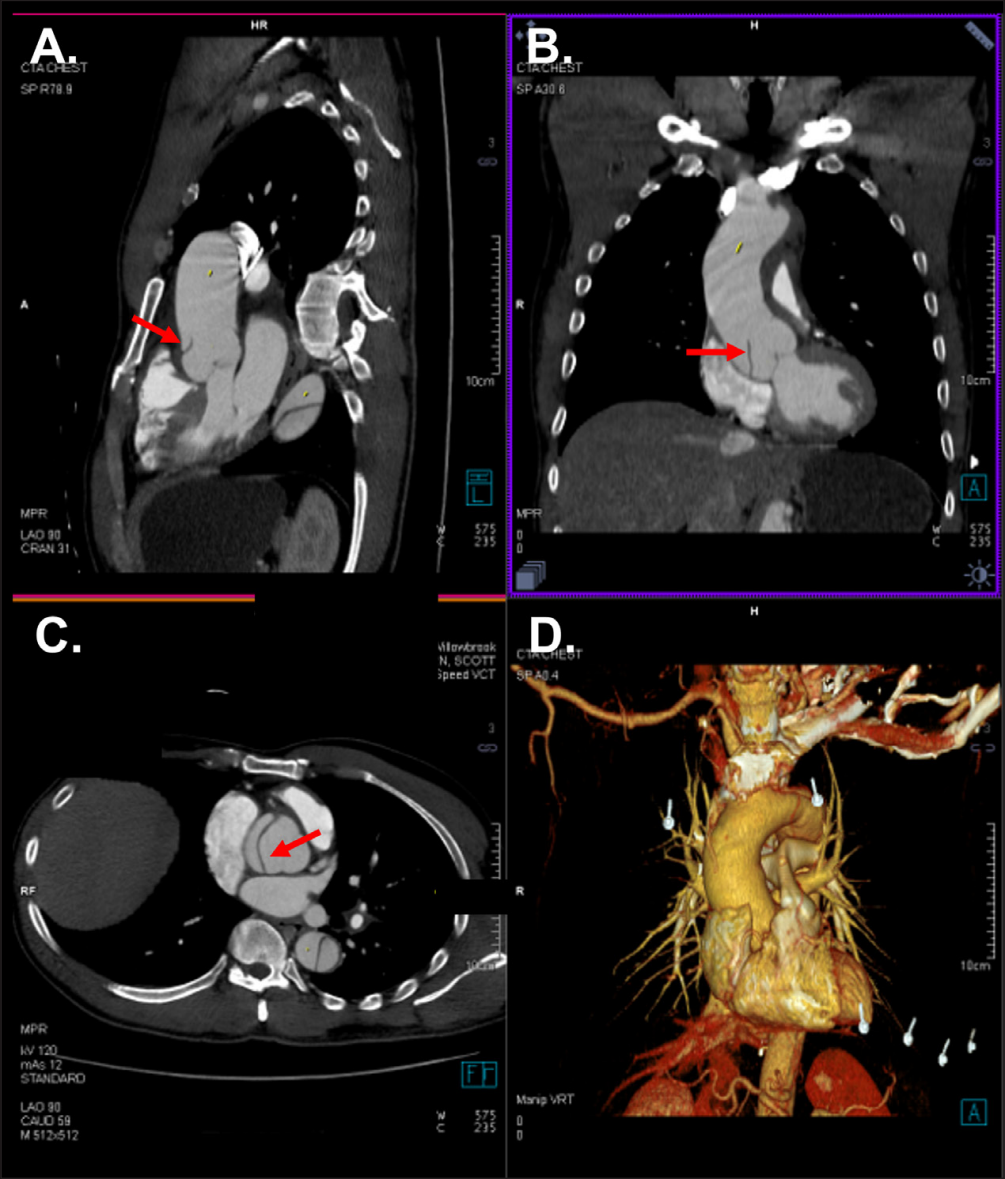
Aortic root dissection. Sagittal **(A)**, coronal **(B)**, and transverse **(C)** computed tomography showing dissection flap in the aortic root (arrow). Three-dimensional reconstructed computed tomography is provided **(D)**.

**Figure 2:**
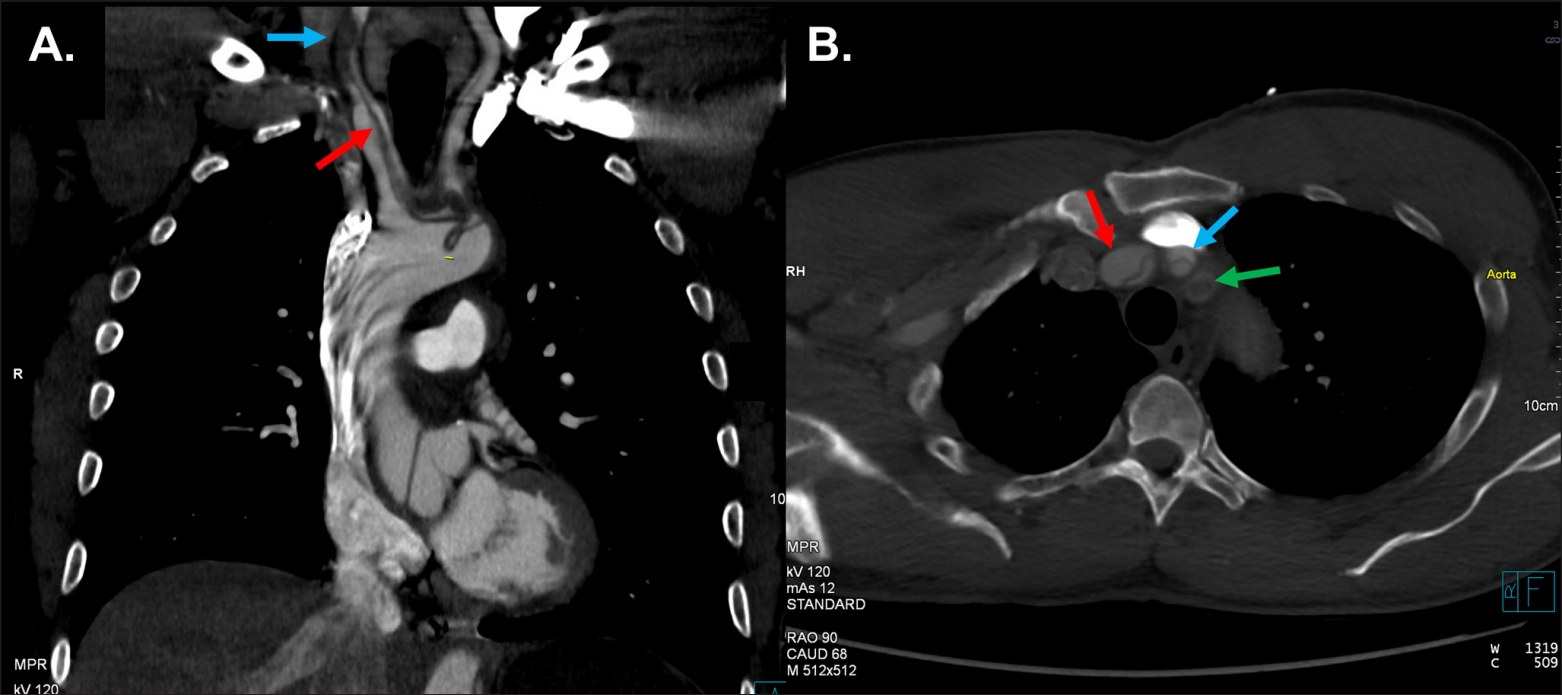
Supra-aortic vessel dissection. Sagittal **(A)** and transverse **(B)** computed tomography showing brachiocephalic artery dissection (red arrow) and near complete occlusion of the right common carotid artery (blue arrow) due to thrombosis of the false lumen. Dissection of the right subclavian artery (green arrow) is also seen.

**Figure 3:**
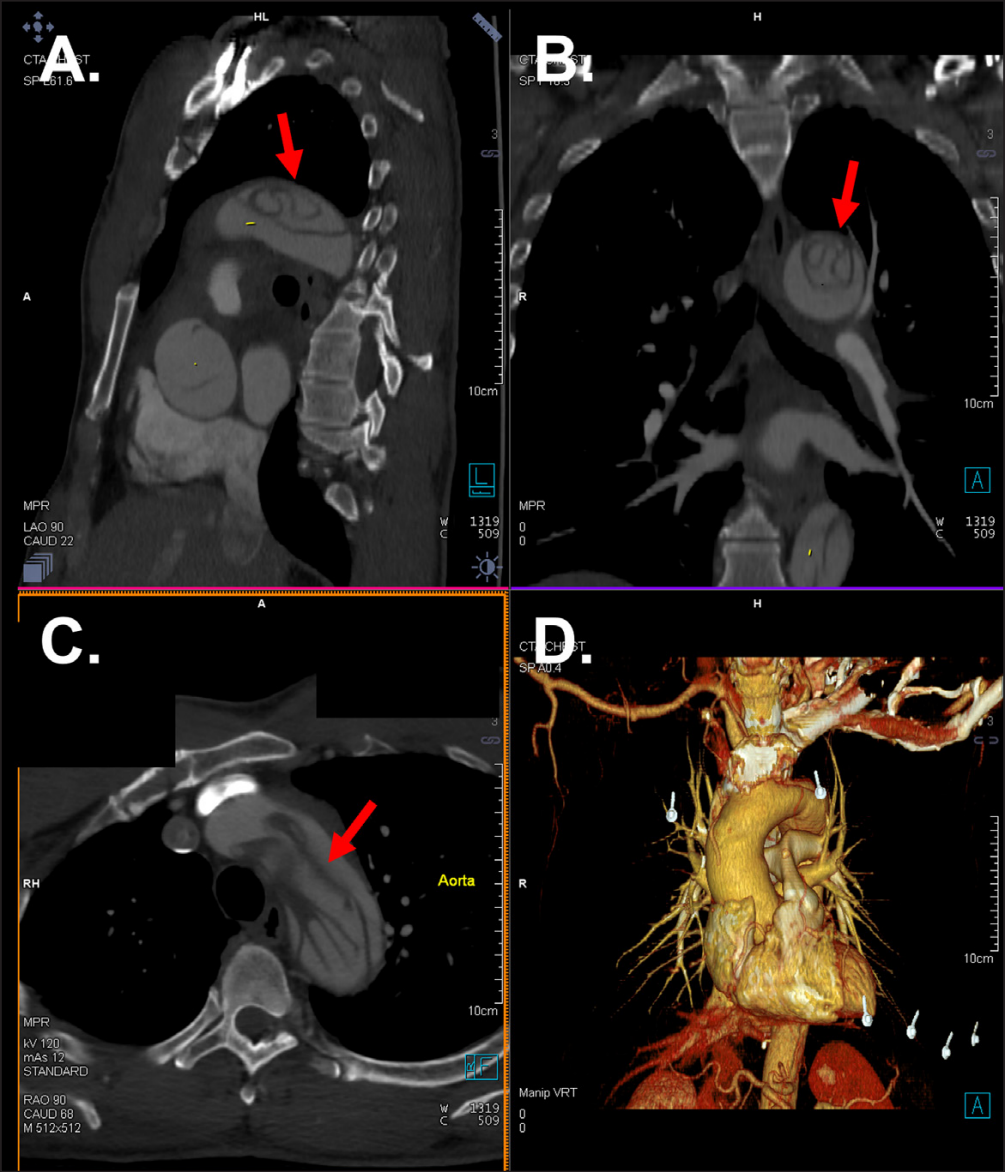
Aortic arch dissection. Sagittal **(A)**, coronal **(B)**, and transverse **(C)** computed tomography showing complex dissection flap at the level of the aortic arch (arrow). Three-dimensional reconstructed computed tomography is provided **(D)**.

Computed tomography angiography revealed aneurysmal dilatation of the aorta with TAAD involving the ascending aorta (4.6 cm), aortic arch, arch vessels, and descending thoracic aorta. A well-defined dissection flap was noted in the aortic root with no extension into the coronary ostia (Figure 1). No intimal flap could be seen in the ascending aorta. The dissection extended into the brachiocephalic artery (BCA), right subclavian artery (RSA), and right common carotid artery (RCCA) with high-grade stenosis and near occlusion due to thrombosis of the false lumen but with some residual flow through the true lumen (Figure 2). The dissection further involved the ostium of the left common carotid artery (LCCA) with effacement of much of the true lumen as well as the ostium of the left subclavian artery (LSA), which had high-grade stenosis and residual flow through the true lumen. The dissection in the left subclavian artery stopped proximal to the vertebral artery and internal mammary artery origins. Marked irregularity of the aortic arch was noted with central displacement, narrowed true lumen, and redundancy of the intima (Figure 3). Multiple fenestrations of the dissection were noted through the descending thoracic aorta, with extension of the dissection into the upper abdomen and differential involvement of the renal arteries as well as near-complete effacement of the true lumen in the infrarenal aorta. Associated mediastinal hematoma at the aortic root level encircled the pulmonary artery and extended to the left hilum, without hemopericardium or tamponade.

The patient’s left-sided hemiparesis was concerning for ischemia secondary to RCCA dissection. Upon arrival, the patient was alert and oriented but reported acute chest pain, abdominal pain, and left-sided paresthesia. Physical examination revealed nonpalpable radial, femoral, and pedal pulses on his left extremities. He was taken immediately to the operating room for TAAD repair.

## Management

An 8-mm Dacron graft was sutured end-to-side to the right axillary artery for arterial inflow. A median sternotomy was performed. A pericardial cradle was created, the patient was fully heparinized, and single cannulation of the right atrium with a dual stage cannula was accomplished. A left atrial sump was placed in the right superior pulmonary vein, and cardiopulmonary bypass with cooling commenced. Profound hypothermia to a core temperature of 22°C was completed. Circulatory arrest was initiated at this point and the ascending aorta opened. No intima could be seen in the ascending aorta. Inspection of the arch revealed the intimal had intussuscepted on itself into the distal arch/descending thoracic aorta. The intussuscepted intima was visualized, grabbed with a long clamp, and rolled back onto itself (Video 1).

**Video 1:** Surgical video of the intussuscepted intima being grasped within the distal arch/descending aorta and rolled back on itself for realignment; see also at https://youtube.com/shorts/f4mnD3ucmgY.

We then divided the aorta from the base of the BCA to the hemiarch. The arch itself appeared to be without tears at the intima. The distal anastomosis was done under circulatory arrest with a 30-mm Dacron graft (DuPont). Once complete, the graft was clamped and flow was established with warming. Total circulatory arrest time was 12 minutes. We then turned our attention to resuspension of the aortic valve using 4-0 prolene suture with pledgets just above the commissural posts. The Dacron graft was cut to length and sutured proximally with 4-0 prolene and pledgets for reinforcement. After warming, the patient weaned easily from cardiopulmonary bypass and was decannulated in a routine fashion. Postoperative hemodynamics were excellent, and the patient was discharged home on postoperative day 5. Follow-up CT angiography at 1 month revealed residual dissection beyond the repair.

## Discussion

Since its first report in 1962, circumferential aortic dissection with intimointimal intussusception remains a rare event, comprising less than 2% of TAADs.^4^ While uncommon, intimointimal intussusception has the potential for lethal complications that depend on the direction of the mobile intimal flap prolapse. If the prolapse occurs retrograde into the left ventricular outflow tract during diastole, it can lead to rapid hemodynamic deterioration via severe aortic regurgitation or obstruction of the coronary ostia.^4^ Similarly, the flap may prolapse distally into the aortic arch. Obstruction of the branching vessels then leads to symptoms of cerebral malperfusion.^5^ In our case, TAAD involved the branching vessels with concerns for cerebral ischemia secondary to RCCA dissection. Recognition of distal intussusception and return of the intima to a normal position is required for a successful repair.

Rapid diagnosis of intimointimal intussusception can be a challenge. Symptoms may mimic other conditions, such as acute coronary syndrome.^5^ If the intimal flap obstructs the coronary ostia, coronary angiography may be difficult.^5^ Angiography and CT are useful for the detection of aortic dissection; however, the intimal flap may be difficult to distinguish from the aortic valve in cases of retrograde prolapse.^5^ TEE is the most reliable imaging modality for intimointimal intussusception because it allows for continuous, real-time visualization of the flap and aortic valve.^4^ In this case, TAAD and intimal flap were successfully visualized with CT, allowing for quick diagnosis and surgical repair and avoidance of lethal complications associated with intimal flap obstruction of nearby arteries.

## Conclusion

Circumferential aortic dissection with intimointimal intussusception is a rare but lethal condition that should be considered when patients present with symptomatic TAAD. The condition may elude diagnosis with angiography or CT but can be reliably visualized with TEE. Emergent open surgical repair of the aorta and aortic valve is necessary, and recognition of distal intimal intussusception with realignment of the intima to a normal position is required for a successful repair.
